# Interleukin-1 beta and tumor necrosis factor alpha inhibit migration activity of chondrogenic progenitor cells from non-fibrillated osteoarthritic cartilage

**DOI:** 10.1186/ar4299

**Published:** 2013-09-13

**Authors:** Helga Joos, Anja Wildner, Cathrin Hogrefe, Heiko Reichel, Rolf E Brenner

**Affiliations:** 1 Division for Biochemistry of Joint and Connective Tissue Diseases, Department of Orthopedics, University of Ulm, Ulm, Germany; 2 Department of Orthopedics, University of Ulm, Ulm, Germany

**Keywords:** Chondrogenic progenitor cells (CPC), migration, IL-1β, TNF-α, cartilage repair

## Abstract

**Introduction:**

The repair capability of traumatized articular cartilage is highly limited so that joint injuries often lead to osteoarthritis. Migratory chondrogenic progenitor cells (CPC) might represent a target cell population for *in situ *regeneration. This study aims to clarify, whether 1) CPC are present in regions of macroscopically intact cartilage from human osteoarthritic joints, 2) CPC migration is stimulated by single growth factors and the cocktail of factors released from traumatized cartilage and 3) CPC migration is influenced by cytokines present in traumatized joints.

**Methods:**

We characterized the cells growing out from macroscopically intact human osteoarthritic cartilage using a panel of positive and negative surface markers and analyzed their differentiation capacity. The migratory response to platelet-derived growth factor (PDGF)-BB, insulin-like growth factor 1 (IGF-1), supernatants obtained from *in vitro *traumatized cartilage and interleukin-1 beta (IL-1β) as well as tumor necrosis factor alpha (TNF-α) were tested with a modified Boyden chamber assay. The influence of IL-1β and TNF-α was additionally examined by scratch assays and outgrowth experiments.

**Results:**

A comparison of 25 quadruplicate marker combinations in CPC and bone-marrow derived mesenchymal stromal cells showed a similar expression profile. CPC cultures had the potential for adipogenic, osteogenic and chondrogenic differentiation. PDGF-BB and IGF-1, such as the supernatant from traumatized cartilage, induced a significant site-directed migratory response. IL-1β and TNF-α significantly reduced basal cell migration and abrogated the stimulative effect of the growth factors and the trauma supernatant. Both cytokines also inhibited cell migration in the scratch assay and primary outgrowth of CPC from cartilage tissue. In contrast, the cytokine IL-6, which is present in trauma supernatant, did not affect growth factor induced migration of CPC.

**Conclusion:**

These results indicate that traumatized cartilage releases chemoattractive factors for CPC but IL-1β and TNF-α inhibit their migratory activity which might contribute to the low regenerative potential of cartilage *in vivo*.

## Introduction

Traumatic injuries of articular cartilage induce pathogenetic processes like chondrocyte death, matrix degradation and release of proinflammatory mediators [1], and represent a major risk factor for the development of osteoarthritis. Current surgical treatment options for cartilage defects include microfracturing [2] and Pridie drilling [3], which enable influx of blood and multipotent mesenchymal stromal cells (MSC) from bone marrow, and frequently end up in fibrocartilage, representing a functionally inferior repair tissue. Strategies to improve local recruitment of bone-marrow-derived MSC into three-dimensional matrices are based on the migratory potential of progenitor cells capable for chondrogenic differentiation. An example already used in the clinic is the autologous matrix induced chondrogenesis (AMIC), which combines microfracturing and a scaffold for ingrowth of bone-marrow-derived MSC [4]. Such an approach could possibly be enhanced by incorporation and controlled release of chemoattractive factors for MSC. Since classical chemokines induce parallel recruitment of inflammatory cells the application of chemoattractive growth factors may be most promising. In the context of cartilage repair the chemoattractive properties of platelet derived growth factor isoforms (PDGF), insulin like growth factor 1 (IGF-1), basic fibroblast growth factor (bFGF), bone morphogenetic proteins (BMPs) or transforming growth factor beta 1 (TGF-β 1) for bone-marrow-derived MSC could be of special interest [5-9]. However, it has been reported that subchondral drilling leads to long-lasting alterations in microarchitecture and bone mineral density of subchondral bone as well as formation of intralesional osteophyts [10]. Therefore, in the case of partial size defects, strategies to recruit CPC from other tissue sources of a joint could be advantageous.

Besides bone marrow and trabecular bone [11], MSC-like cells have been identified in synovial membrane [12], synovial fluid [13,14], infrapatellar fat [15] and articular cartilage itself [16-18]. These cell populations are not identical but they fulfill a set of minimal criteria proposed by the Mesenchymal and Tissue Stem Cell Committee of the International Society for Cellular Therapy (ISCT) to define human MSC [19]. Besides the adherence to plastic, the expression of specific surface antigens is an important criterion. As there is no single specific MSC marker, a combination of positive and negative surface markers are used to define MSC. According to ISCT, the minimal panel of markers includes CD105, CD73 and CD90 but excludes the hematopoietic markers CD45, CD34, CD14 (or CD11b), CD19 (or CD79α) and HLA class II [19]. Various additional positive and negative surface markers, including Stro-1, MSCA-1, CD166, CD44, CD90, CD29, CD54, CD9, CD146 and CD133, have been described [15,20,21], which may help to develop a cell-surface antigen profile for identification of MSC subpopulations. The third criterion is the ability of MSC to differentiate *in vitro *under lineage-specific culture conditions into osteoblasts, adipocytes and chondrocytes first described by Pittenger *et al*. [22].

The first studies on the presence of MSC-like cells in normal and osteoarthritic human cartilage were based on the characterization of enzymatically released cells [16-18]. Recently, in digests of full thickness normal human cartilage a progenitor cell population has been identified based on the expression of α5-integrin and characterized on clonal level [23]. Moreover, CPC were described in the superficial layer of articular cartilage in young calves [24]. It remained open whether cells with a chondroprogenitor phenotype are able to leave their local niche in cartilage actively. More recently, migratory CPC have been described in late-stage human osteoarthritic cartilage characterized by surface fissures and cell clusters [25]. They grew out of tissue slices and may be derived from breaks in the tidemark, express several suface markers present on bone marrow derived MSC and respond to serum or TGF-β3 by an increase in migratory activity [25]. Moreover, Seol *et al*. recently showed a repopulation of nonviable areas in damaged cartilage by CPC derived from surrounding tissue in a bovine *in vitro *cartilage trauma model [26]. Therefore, CPC are discussed as a progenitor cell population suitable for cartilage regeneration and some modulations of CPC through application of growth factors, RUNX-2 knockdown or contemporary anti-inflammatory therapy have been suggested to enhance their regenerative potential [27].

From this state of knowledge the questions arise whether (i) migratory CPC are present in human cartilage tissue representing an early stage of degeneration, (ii) blunt traumatization of cartilage that is associated with a loss of viable chondrocytes results in the release of chemoattractive factors for human CPC and (iii) a local pro-inflammatory milieu as observed after major joint injuries influences their migratory potential. The proinflammatory cytokine IL-1β, for example, may also have negative effects on chondrogenic differentiation as the inhibition of IL-1β-induced activation of NF-κB was shown to facilitate chondrogenesis in a canine MSC differentiation model [28]. Therefore, the aims of the present study were to identify and characterize mesenchymal stromal cells established by spontanous outgrowth from macroscopically intact, non-fibrillated osteoarthritic cartilage with a smooth surface, which can be distinguished from ulcerated damaged tissue in osteoarthritis [29]. The migration activity of the outgrown cells was analyzed in response to distinct growth factors (IGF-1 and PDGF-BB) as well as the cocktail of factors released from human cartilage after *in vitro *traumatization. Furthermore, the influence of IL-1β and TNF-α as the major pro-inflammatory cytokines released within the joint after trauma and involved in the initiation and progression of degenerative joint disease was investigated.

Our results show that macroscopically intact osteoarthritic cartilage contains migratory cells with a chondroprogenitor phenotype and a very similar surface marker profile compared to bone-marrow-derived MSC. Besides single growth factors like IGF-1 and PDGF-BB, the combination of substances released by blunt traumatized cartilage effectively induced directed cell migration of CPCs but the addition of IL-1β or TNF-α had detrimental effects on migratory activity. This indicates that inflammatory processes may have profound influence on local chondroprogenitor cell recruitment for endogenous repair of blunt cartilage injuries associated with a loss of viable cells or *in situ *regeneration of partial thickness cartilage defects.

## Methods

### Cell isolation, cell culture and determination of outgrowth activity

Multipotent MSC and CPC were isolated during routine surgical procedures with informed consent of the patients and in accordance with the terms of the Ethics Committee of the University Ulm.

MSC were isolated from human bone marrow aspirates (mean age 27, range 14 to 45 years) using a density gradient centrifugation (Biocoll separation solution: Biochrom Seromed, Berlin, Germany) as described previously [7], and cultured in basal medium consisting of DMEM with 10% fetal calf serum (FCS), 2 mM L-glutamine, and 100 U/ml penicillin/streptomycin (all Biochrom Seromed, Berlin, Germany) at 37°C, 5% CO_2 _in 95% humidity. Cells were split at a confluence of 80%.

CPC were obtained from the resected tissue of 71 patients with osteoarthritis undergoing total knee replacement (mean age 67, range 45 to 87 years) according to Koelling *et al*. [25]. In brief, cartilage slices with an edge length of about 4 mm were cut from macroscopically intact, non-fibrillated regions and placed in a culture dish with basal medium. Patients with systemic inflammatory diseases, such as rheumatoid arthritis or spondylarthropathies, were excluded. After outgrowth from cartilage tissue, CPC were expanded and cultured like MSC. For cytokine studies, 1 ng/ml IL-1β and 10 ng/ml TNFα (both tebu-bio, Offenbach, Germany), respectively, were added to the medium and renewed every two to three days. To determine the outgrowth activity after 14 days of cultivation, emigrated cells were trypsinized after the removal of cartilage slices and counted by trypan blue exclusion staining using a Neubauer chamber.

### Immunocytochemistry

To analyze freshly emigrated CPC, cartilage slices were directly placed in four-chamber culture slides (BD Pharmingen, San Diego, CA, USA). After outgrowth, the tissue slices were removed and the CPC used for immunocytology. Otherwise, CPC were cultured on culture slides in passage 3 or 4. The cells were washed and fixed with 4% formaldehyde, incubated with primary antibodies overnight at 4°C (CD90, CD54, CD166: Acris, Hiddenhausen Germany; CD73: AbD Serotec, Düsseldorf, Germany; Cadherin: Life Technologies, Darmstadt, Germany) and stained with the DAKO LSAB™2 Kit (Hamburg, Germany). The cytoskeleton of CPC was visualized by actin-labeling with Phalloidin-FITC (Sigma-Aldrich, Taufkirchen, Germany), Vinculin-labeling with a specific primary antibody (Sigma-Aldrich) and a secondary Alexa Fluor 488-coupled antibody (Life Technologies) and counterstained with DAPI (Sigma).

### Flow cytometry analysis

Cultured cells in passage 3 or 4 were detached using a trypsin/EDTA solution (Biochrom Seromed, Berlin, Germany) and incubated with antibodies against the surface markers and the species-matched isotype controls for 30 minutes in the dark on ice. In the case of biotinylated antibodies (CD44 Biotin), cells were washed with PBS before adding PerCP Streptavidin. The samples were characterized by single- and four-color immunofluorescence and 1 × 10^4 ^cells were analyzed on a Becton Dickinson FACSCalibur flow cytometer (BD Biosciences, Heidelberg, Germany) with dual-laser technology. The CellQuest software V. 5 from Becton Dickinson was used for analysis. The amount of positive cells was calculated as a percentage in comparison to the isotype control. A maximum of 1% positive cells by staining with the isotype control were allowed. CD146 and IgG2a were obtained from R&D Systems (Minneapolis, MN, USA), CD133 was obtained from Miltenyi Biotec (Bergisch Gladbach, Germany) and MSCA-1, Stro-1 and IgM were obtained from BioLegend (San Diego, CA, USA). All other antibodies were provided by BD Pharmingen (Heidelberg, Germany).

### Differentiation assays

After cultivation of CPC in basal medium until 80% confluence, cells were cultivated in differentiation media or basal medium for control for up to four weeks as described for MSC [17,30]. The adipogenic medium consisted of basal medium with 0.5 μmol/l dexamethasone, 0.5 μmol/l isobutylmethylxanthine and 50 μmol/l indomethacin. The osteogenic medium consisted of basal medium with 0.1 μmol/l dexamethasone, 50 μg/ml ascorbic acid and 200 μg/ml β-glycerophosphate. For chondrogenic differentiation in micromass culture, 2 × 10^5 ^CPC were pelleted by centrifugation and cultivated in chondrogenic medium consisting of DMEM and Ham's F-12 with 4.5 g/l glucose, 100 U/ml penicillin/streptomycin, 40 ng/ml L-proline, 0,1 μmol/l dexamethasone, 50 μg/ml ascorbic acid supplemented with TGF-β3 (10 ng/ml), BMP-6 (10 ng/ml) and 10 μl/ml ITS+ (Sigma). The differentiation was confirmed by histological staining.

The von Kossa and Oil Red staining was performed as previously described [17]. Alkaline phosphatase activity was estimated with the Leukocyte Alkaline Phosphatase Kit (Sigma-Aldrich, Taufkirchen, Germany) as prescribed by the manufacturer. The immunostaining was performed on paraffin-embedded tissue sections with collagen II antibody (Acris, Hiddenhausen, Germany) and cartilage oligomeric matrix protein (COMP) antibody (kindly provided by Dr. Zaucke, Köln, Germany) and the DAKO LSAB™2 Kit (Hamburg, Germany).

### Chemotaxis and attachment assay, growth factors, cytokines and supernatant of cartilage tissue culture

Chemotaxis assays were performed with recombinant human (rh) PDGF-BB (BioLegend, Fell, Germany, 10 ng/ml), rhIGF-1 (Biomol, Hamburg, Germany, 100 ng/ml), IL-1β (tebu-bio, 0.1 to 10 ng/ml), TNFα (tebu-bio, 1 to 100 ng/ml) and IL-6 (Biomol, 1 ng/ml). Additionally, serumfree supernatant of untraumatized and traumatized cartilage explants of four donors (mean age 66, range 62 to 70 years) was used. The tissue was prepared and treated as described elsewhere [31]. In brief, full-thickness explants were harvested from well-preserved cartilage of donors undergoing total knee joint replacement due to osteoarthritis. The cartilage was traumatized by an impact load of 0.59 J using a drop-tower device and cultivated in serum-free medium for 24 h, unimpacted explants of the same patients served as control. The supernatant was harvested and stored at -80°C until use.

Cell migration was analyzed by a modified Boyden chamber assay, using a 48-well microchemotaxis chamber (NeuroProbe Inc., Baltimore, MD, USA) with polycarbonate filters with 8 μm pores (NeuroProbe Inc.) as previously described [5]. MSC and CPC were trypsinized and suspended in serum-free DMEM. Growth factors were diluted in serum-free DMEM, filled into the lower compartment of the chemotaxis chamber and covered with the chemotaxis filter. The upper wells were loaded with 50 μl cell suspension (1 × 10^4 ^cells) and incubated for four hours at 37°C, 5% CO_2 _in 95% humidity. The filter was taken off, washed with PBS and scraped with a rubber wiper to remove the non-migrated cells on the upper side of the filter. The migrated cells on the lower side were fixed with 4% formaldehyde, stained with Giemsa solution (Merck, Darmstadt, Germany) and counted. DMEM in the lower well served as a negative control (basal migration) for each experiment. The results were determined as the total number of migrated cells.

To distinguish chemotaxis from undirected chemokinesis migration analyses with the growth factors, supernatants of cartilage tissue and cytokines were performed in the presence and absence of a concentration gradient [32].

For the attachment assay on the polycarbonate filters, 5 × 10^2 ^CPC in 50 μl serum-free DMEM were given into the upper well of the Boyden chamber and incubated for 30 and 60 minutes. Adherent cells on the upper side of the filter were fixed, stained and counted as described above.

### Scratch assay

CPC were grown to a confluent monolayer. After making a "scratch" in the cell layer with a 200 μl pipette tip, CPC cultures were rinsed gently to discard debris. The cells were cultivated in basal medium with or without cytokines (1 ng/ml IL-1β and 10 ng/ml TNFα, respectively) for 48 h, the medium was renewed after 24 h. Migration was documented by phase contrast microscopy.

### Statistical analysis

All experiments were analyzed using GraphPad Prism version 5.0d (GraphPad Software, San Diego, CA, USA). For statistical analysis a Student's *t*-test or one-way ANOVA was used with Bonferroni multiple comparison post-test. A *P *< 0.05 was considered significant (*). Other levels of significance were defined as follows: *P *< 0.01 (**) and *P *< 0.001 (***).

## Results

**Figure 1:**
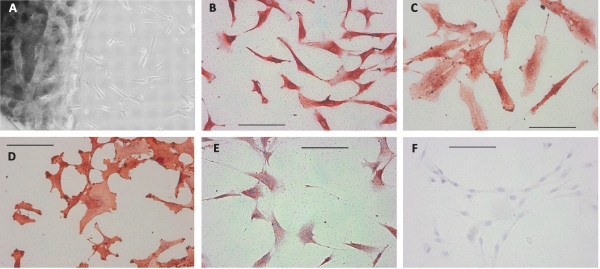
**Freshly outgrown CPC were characterized morphologically and by immunocytochemical stainings**. **A: **Cartilage slice after 11 days of cultivation with emigrated cells of fibroblastoid appearence. **B-F: **Immunocytochemical staining of chondrogenic progenitor cells (CPC) specific for CD73 (B), CD90 (C), CD166 (D) and CD54 (E); F: negative control. The bar indicates 100 μm.

CPC cultures could be established by spontanous outgrowth from macroscopically intact cartilage of 97% of patients with knee osteoarthritis. The first cells were visible after 5 to 10 days and they had a fibroblastoid morphology. Figure 1 depicts freshly emigrated CPC (A) and their positive staining for the typical MSC surface markers CD73, CD90, CD166 and CD54 (B-F) in immunocytochemistry.

### Characterization of MSC and CPC by immunocytology

Due to the lack of a specific single surface marker protein for MSC, we used variable combinations of different markers for flow cytometric analysis to characterize the isolated MSC and CPC. MSC from bone marrow and CPC grown out from cartilage were stained with single markers (see Figure S1 in Additional file 1). CPC expressed similar levels of CD29, CD44, CD73, CD90 (all >90%), CD105 and CD166 (>75%), CD54 and MSCA-1 (44 to 66%), Stro-1 (16 to 23%) and CD88 (9 to 10%) as bone marrow-MSC. Significantly different levels of expression were only observed for CD9 (MSC: 46.7 ± 4.3% mean ± standard error of positive cells, CPC: 80.7 ± 3.5%) and CD146 (MSC: 44.0 ± 6.8%, CPC: 11.9 ± 2.3%). CPC and MSC were negative for the hematopoietic stem cell markers CD34 and CD133, the lymphocyte marker CD45 and Octamer-binding transcription factor (Oct-3/4). CD14 and CXC chemokine receptor 4 (CD184) were only slightly expressed (<4%).

**Figure 2:**
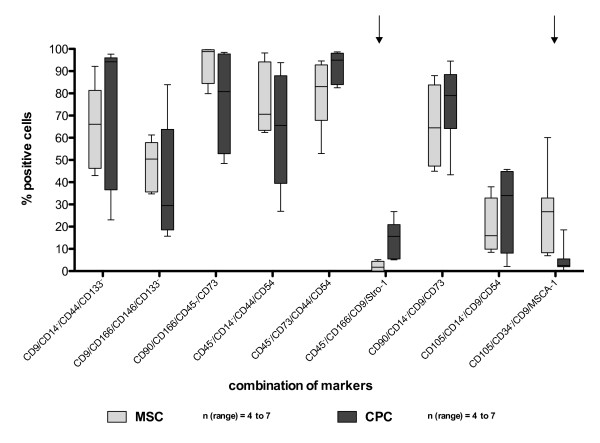
**Marker expression profiles from MSC and CPC**. Both cell types were respectively stained with four markers in different combinations. The percentage of quadruple stained cells is depicted on the y-axis. Due to the varying patient collectives the number of different donors is declared as n (range). The arrows highlight the marker combinations which showed significance in Student's *t*-test: CD45^-^/CD166/CD9/Stro-1 (*P *= 0.003), CD105/CD34^-^/CD9/MSCA-1 (*P *= 0.019). CPC, chondrogenic progenitor cells; MSC, mesenchymal stromal cells.

A combination of the above-mentioned markers was used to characterize both cell types in more detail. We investigated 25 different quadruplicate marker combinations (Figure 2 and Figure S2 in Additional file 2) and compared the expression profiles between MSC and CPC. Statistical analysis showed significant differences in two marker combinations (Figure 2; arrows): 2.14 ± 0.8% of the MSC and 14.62 ± 7.1% of the CPC expressed CD45^-^/CD166/CD9/Stro-1 (*P *= 0.003; n ≥ 6) and 25.27 ± 7.1% of the MSC and 4.95 ± 2.3% of the CPC expressed CD105/CD34^-^/CD9/MSCA-1 (*P *= 0.016; *n *= 7). The other 23 combinations showed no significant differences between MSC and CPC.

A characterization of CPC concerning adhesion receptors and the actin cytoskeleton revealed a strong expression of the integrin subunits α1, α5 and β1 (>80% positively stained cells, α1 and α5: see Figure S3A in Additional file 3; β1 (CD29): see Figure S1 in Additional file 1) and a partial expression of α2 and α6 (8.5% and 23.9% positively stained cells, respectively, see Figure S3A in Additional file 3) in flow cytometry. CPC cultured in monolayer stained positive for Cadherin 11 and showed the formation of actin stress fibers as well as vinculin-staining at focal adhesion sites in immunocytochemical staining (Figure S3B, C in Additional file 3).

### Differentiation potential of CPC

CPC were seeded either in monolayer (adipogenic, osteogenic) or 3D micromass culture (chondrogenic) to examine their multipotent differentiation potential. We induced adipogenesis, osteogenesis and chondrogenesis for four weeks and confirmed the differentiation by PCR gene expression of respective marker genes (LPL/leptin for adipogenic, Runx2/ALPL/osteocalcin for osteogenic, SOX9/COL2A1/ACAN/COMP/COL10A1 for chondrogenic differentiation, see Figure S4 in Additional file 4) and histological staining. Cells cultured in basal medium over four weeks were used as control.

**Figure 3:**
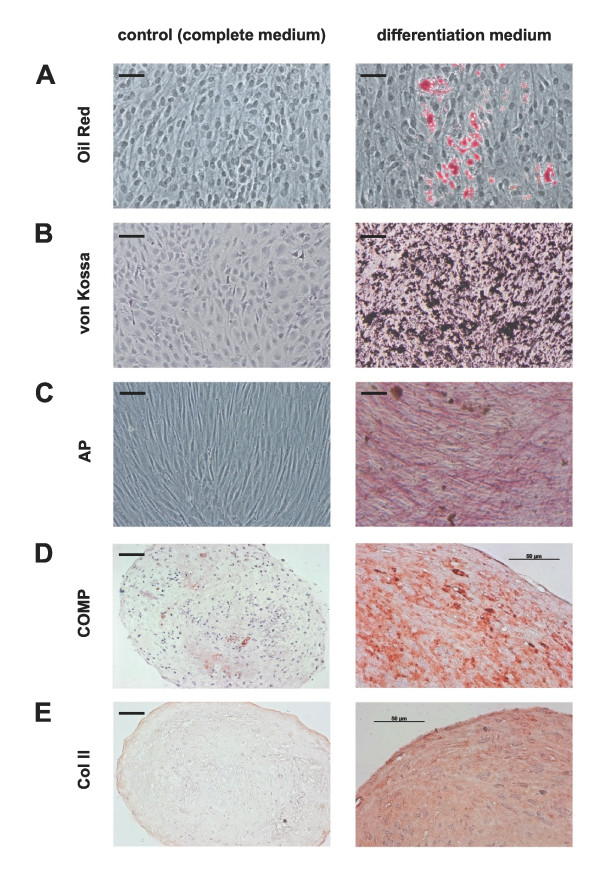
**Differentiation potential of CPC**. Cells were cultured with basal medium as control (left column) or with differentiation medium (adipogenic, osteogenic, chondrogenic) for up to four weeks. Chondrogenic progenitor cells (CPC) are **A: **Oil Red O positive after adipogenic differentiation (red: stained lipid droplets) and positive after osteogenic differentiation for **B: **von Kossa (black: mineral deposition) and **C: **alkaline phosphatase (AP) activity (red dye deposit). The chondrogenic differentiation was performed as 3D micromass culture (**D-E**). Immunohistochemical staining with cartilage oligomeric matrix protein (COMP; D) and collagen II (Col II; E) were positive. The bar indicates 100 μm unless otherwise noted.

The adipogenic differentiation of CPC is shown in Figure 3A. We could observe the formation of cell colonies that contained lipid vacuoles which were positive for Oil Red O. The majority of cells, however, did not develop lipid droplets comparable to the negative control cultivated with basal medium. CPC showed adipogenic differentiation to a lesser extent than MSC analyzed in parallel (data not shown), indicating that only a subpopulation of the CPC exhibits adipogenic potency.

CPC were also cultured for four weeks with osteogenic medium and were stained with von Kossa and for alkaline phosphatase to investigate the osteogenic potential. Figure 3B shows black calcium deposits after von Kossa staining. Further staining for AP (alkaline phosphatase), one of the early marker in osteogenesis, showed in the presence of osteogenic supplements diffuse, red dye deposit as a measurement of AP activity (Figure 3C). In control wells these deposits could not be detected. Gene expression of osteocalcin was strong after osteogenic differentiation, but could as well be weakly detected in undifferentiated cells (Figure S4 in Additional file 4). A faint staining of osteocalcin could partly be detected in undifferentiated CPC in monolayer by immunocytology (data not shown).

To analyze if CPC can be differentiated into chondrocytes, cultivation was performed as micromass pellet culture. After cultivation with chondrogenic medium over four weeks, the matrix stained positive for COMP (Figure 3D) and collagen type II (Figure 3E) indicating chondrogenic differentiation. In the absence of chondrogenic factors, there was no positive staining for these matrix proteins of cartilage.

### Chemotaxis assay

**Figure 4:**
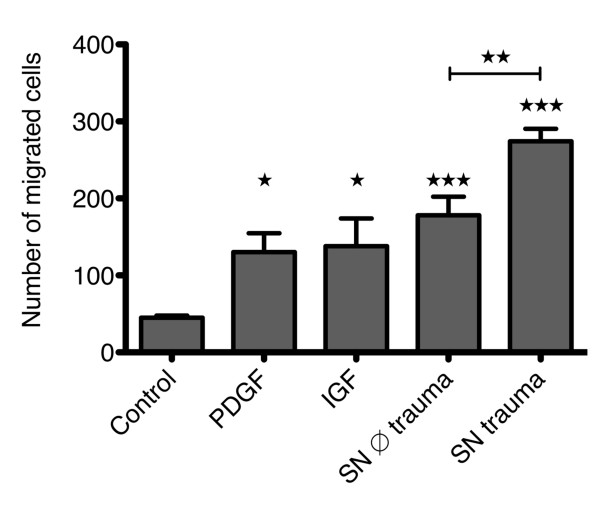
**Effects of PDGF, IGF and cartilage supernatant on CPC migration**. The number of migrated cells without stimulation and stimulated with platelet-derived growth factor (PDGF) (10 ng/ml), IGF (100 ng/ml) or supernatant of unimpacted cartilage (supernatant (SN) ∅ trauma) and impacted cartilage (SN trauma), respectively, are depicted. The values represent the mean ± SEM with n ≥3. Statistical significance was evaluated by ANOVA followed by a Bonferroni multiple comparison post-test with *P < 0.05, **P < 0.01 and ***P < 0.001.

Cell migration of CPC was significantly stimulated by 10 ng/ml PDGF-BB and 100 ng/ml IGF-1 as shown in Figure 4. Furthermore, serum-free conditioned medium of human osteoarthritic cartilage samples significantly stimulated CPC migration. Supernatant from cartilage injured *in vitro *by a defined blunt trauma led to a further significant increase in the number of migrated cells (Figure 4). The stimulation of cell migration was only present in case of a concentration gradient (data not shown), indicating that in all cases site-directed migration (chemotaxis) was induced and not random migration (chemokinesis).

**Figure 5:**
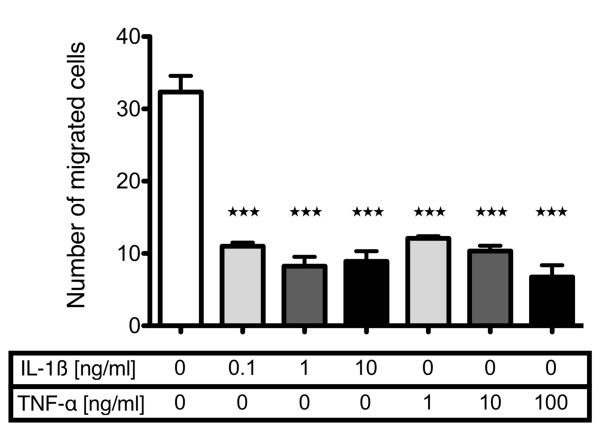
**Dose-dependent effects of IL-1β and TNF-α on basal migration of CPC**. Cells were analyzed in basal medium with and without addition of interleukin (IL)-1β and tumor necrosis factor (TNF)-α in the indicated concentrations. The values represent the mean ± SEM with n ≥3. Statistical significance was evaluated by ANOVA followed by a Bonferroni multiple comparison post-test with *P < 0.05, **P < 0.01 and ***P < 0.001. CPC, chondrogenic progenitor cells.

In contrast to the observed chemotaxis towards PDGF-BB, IGF-1 and the supernatant of traumatized cartilage, a gradient of IL-1β or TNF-α led to profound and highly significant inhibition of CPC-migration over a broad concentration range (IL-1β from 0.1 to 10 ng/ml, TNF-α from 1 to 100 ng/ml) as shown in Figure 5. Exemplarily, it could be shown that both cytokines also inhibited CPC migration when they were present in the upper and lower compartment without concentration gradient (data not presented).

**Figure 6:**
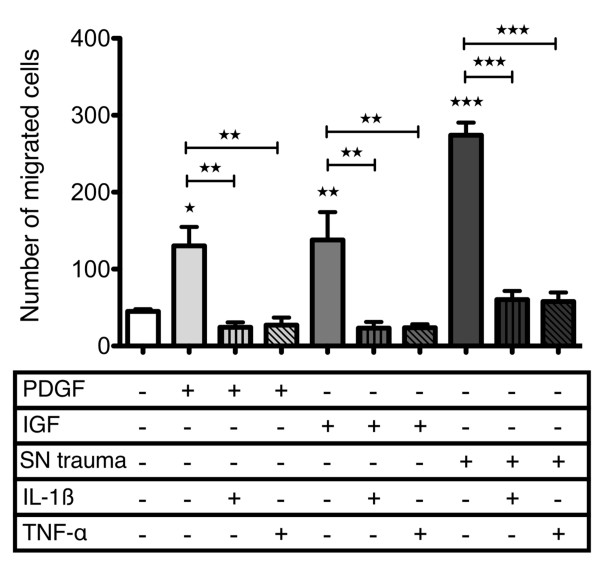
**Effects of IL-1β and TNF-α on stimulated migration of CPC**. Chondrogenic progenitor cells CPC migration was stimulated with platelet-derived growth factor (PDGF) (10 ng/ml), IGF (100 ng/ml) or supernatant of impacted cartilage (supernatant (SN) trauma) and inhibited with interleukin (IL)-1β (1 ng/ml) and tumor necrosis factor (TNF)-α (10 ng/ml), control cells remained unstimulated. The values represent the mean ± SEM with n ≥3. Statistical significance was evaluated by ANOVA followed by a Bonferroni multiple comparison post-test with *P < 0.05, **P < 0.01 and ***P < 0.001.

To test a possible interference with chemoattractive effects of single growth factors, we analyzed the effects of PDGF-BB and IGF-1 without and with parallel gradients of IL-1β and TNF-α. The results clearly showed that 1 ng/ml IL-1β or 10 ng/ml TNF-α completely abrogated the stimulatory effect of these growth factors (Figure 6). An inhibition below basal levels (medium alone) was also found exemplarily when the cytokines were present homogenously in the upper and lower compartment (data not shown). Moreover, the powerful chemotactic effect of the factor cocktail released by *in vitro *traumatized cartilage was significantly inhibited to basal levels, but not below that value as observed with PDGF-BB or IGF-1 co-application (Figure 6). An attachment assay with the migration filters indicated that respective concentrations of IL-1β and TNF-α had no influence on the number of adherent CPC after 30 and 60 minutes (data not shown). IL-6, which is increased (to about 1 ng/ml) in supernatants of traumatized cartilage [31] did not affect basal or PDGF-stimulated CPC migration at 1 ng/ml (Figure S5 in Additional file 5).

### Scratch assay and primary outgrowth in the presence of IL-1β and TNF-alpha

**Figure 7:**
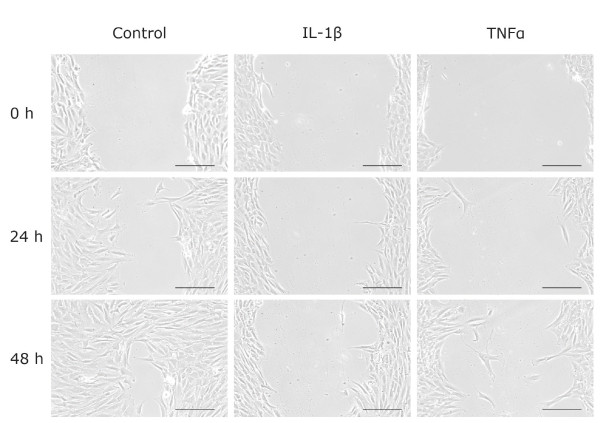
**CPC migration analyzed by scratch assay**. Confluent chondrogenic progenitor cells (CPC) were scratched, incubated with or without interleukin (IL)-1β (1 ng/ml) or tumor necrosis factor (TNF)-α (10 ng/ml) and documented by phase contrast microscopy after the indicated time points. The bars indicate 200 μm. Representative pictures of four independent experiments are shown.

We next performed a commonly used wound-healing assay, also called a scratch assay, to determine the influence of IL-1β and TNF-α on cell migration in an injury-induced environment over a period of 48 h. Figure 7 shows that CPC infiltrated the gap after 24 h and nearly closed it after 48 h under control conditions. In contrast, the addition of 1 ng/ml IL-1β almost abrogated and of 10 ng/ml TNF-α strongly slowed down cell migration in the observation period.

The same cytokines and concentrations were used to investigate the influence of pro-inflammatory stimulation on the primary outgrowth activity of CPC from cartilage slices. The number of outgrown cells after 14 days of cultivation averaged 1,800 cells/well under control conditions whereas addition of IL-1β (1 ng/ml) or TNF-α (10 ng/ml) both reduced outgrowth to less than 10 cells/well on average (Figure S6 in Additional file 6).

## Discussion

In agreement with previous studies we found that human articular cartilage contains a cell population that is characterized by a primary mesenchymal progenitor phenotype or acquires this phenotype during *in vitro *expansion [16-18,23,33,34]. A principle difference from most previous experimental approaches is that we established the cell cultures by active outgrowth and not by enzymatic digestion, which selects for cells with migration potential. This approach was first used by Kölling *et al*. [25], who described migratory CPC in end-stage fibrillated cartilage with breaks in the subchondral bone plate allowing ingrowth of blood vessels. Their observation is in agreement with the hypothesis that the presence of cells with characteristics of MSC in adult vascularized tissues may be based on a common perivascular origin [35]. We identified and characterized outgrowing cells with a CPC phenotype in macroscopically intact cartilage from osteoarthritic joints representing an early stage of tissue degeneration. Although single breaks in the subchondral bone plate cannot be excluded, rather our results support the concept that mesenchymal progenitor cells may be otherwise present in cartilage. The developmental origin of these cells and the influence of the osteoarthritic process still remain to be elucidated. The surface marker profile of outgrowing cells proved to be identical to previous studies on enzymatically digested and culture expanded cells from human articular cartilage [16-18,23] and to cells obtained by outgrowth from severely degenerated osteoarthritic cartilage [25] or normal bovine cartilage [26]. A direct comparison of 18 single surface markers with bone-marrow derived MSC revealed significant differences only for CD9 and CD146, which may be based on replicative senescence of CPC during proliferation after outgrowth since an increase in CD9 and a decrease in CD146 has been observed in higher passages of bone-marrow-derived MSC [36]. CD146 is a surface marker of pericytes and expressed on a population of bone marrow derived subendothelial cells for which true self-renewing capacity was described [37]. Therefore, its lower expression may also relate to the origin from a primary avascular tissue. The surface marker analysis of 25 different quadruplicate marker combinations going far beyond existing data on CPC did not reveal much difference in comparison to bone-marrow-derived MSC, indicating a major overlap of subpopulations. Flow-cytometric integrin analyses showed strong expression of the integrin subunits α5 and β1 and a partial expression of α2 and α6 in accordance with previous studies [25]. The presence of the fibronectin receptor (α5/β1) is in line with the identification of a cartilage progenitor population differentially isolated by fibronectin binding [23]. Differentiation analysis under lineage-specific conditions confirmed the potential for chondrogenic, osteogenic and adipogenic differentiation known from other studies on cartilage derived progenitor cells [16-18,23]. An only partial development of lipid droplets in adipogenic differentiation observed in our study is also described in the literature and may be due to inhibitory factors from preadipocytes [16]. The observation of slight osteocalcin expression in undifferentiated CPC may be ascribed to their osteoarthritic origin [38]. The absence of differentiation and marker gene expression without lineage-specific induction supports the progenitor status of CPC. As described for bone-marrow-derived MSC, chondrogenic differentiation with TGF-β3 was associated with the induction of collagen type × on RNA-level [39], indicating further similarity.

From a clinical point of view, migratory chondroprogenitor cells of cartilage are of interest for novel strategies to improve cell recruitment into cartilage defects without perforation of the subchondral bone plate and to support endogenous repair of blunt injured cartilage as a compensation for trauma-induced chondrocyte loss. Local application of PDGF-BB and IGF-1 has been suggested as a promising strategy for induction of cartilage repair. Positive effects could be expected from stimulation of proliferation as well as proteoglycan synthesis of chondrocytes and reduction of proteoglycan degradation [40]. Furthermore, these growth factors were described to act as an anti-inflammatory agent by inhibiting IL-1β-mediated NF-κB-signalling and apoptosis [41]. We found that both growth factors also stimulated site-directed migration of CPC *in vitro *- as previously reported for bone-marrow derived MSC [5,6]. Possibly this biological functionality may contribute to enhanced repair of cartilage defects *in vivo *by transplantation of chondrocytes overexpressing IGF-1 [42]. The cocktail of factors released from full thickness cartilage explants generated by sharp excision was also chemoattractive for CPC and an additional blunt injury leading to necrosis or apoptosis of about half of the chondrocytes [31] increased this cell-biologic function significantly. In our cartilage trauma model, the cytokine IL-6 is released, leading to a concentration of about 1.8 ng/ml after 24 h [31]. The migration assays with up to 10 ng/ml IL-6, however, neither showed a positive nor negative effect on migratory activity of CPC. A profiling of proteins released by cartilage in response to mechanical compression injury [43,44] identified potential candidates for chemoattractive effects like fibronectin, CTGF and osteopontin, which have been shown to be involved in MSC migration [45-47] and may contribute to the activating effect of the supernatant on CPC in its complex composition. A recent study on bovine cartilage indicated that the release of high mobility group box chromosomal protein 1 (HMGB-1) and receptor for advanced glycation end product (RAGE)-mediated processes partly explains the chemotactic effect [26].

Though CPC have the capacity to actively leave their original niche and to invade cartilage tissue [25], the recruitment of CPC after trauma *in vivo *seems to be low, indicated by reduced cell density at the site of a preceding trauma [48]. An interference of the proinflammatory cytokines IL-1β and TNF-α, which are secreted from synovial cells and increase in synovial fluid early after the injury of a joint [49] could account for this effect. In contrast to IL-6, these cytokines had detrimental effects on CPC migration and abrogated the effect of PDGF-BB, IGF-1, as well as trauma supernatants. In the Boyden chamber assay this effect was present with and without concentration gradient of the cytokines indicating that basic mechanisms of cell migration like adhesion processes, MMP-synthesis and - activation as well as cytoskeletal reorganization may be negatively affected. In the experimental setting, negative effects on CPC adhesion could not be observed. In human chondrocytes, however, it was shown that IL-1β interferes with F-actin structures and other cytoskeletal components [50], which might impair cell motility as the cytoskeleton is known to play a pivotal role in cell movement. The inhibitory effects of IL-1β and TNF-α were confirmed in a scratch assay which allows observation of cell migration in an injury-induced environment [51], supporting the assumption of biological relevance. Finally, the primary outgrowth of CPC from their cartilage origin was markedly impaired by both cytokines, indicating that these pro-inflammatory factors also inhibit the process of release from the native environmental niche which is not defined so far. The migration analyses used covered different time spans from 4 h (Boyden Chamber) to 14 days (outgrowth) which shows that the inhibitory effect is not compensated over an extended period. These findings may also suggest a role for IL-1β and TNF-α in osteoarthritis that was unaccounted for so far. Besides promoting the phenotypic shift of chondrocytes and cartilage destruction [52], an impairment of CPC recruitment could compromise the ability of endogenous repair.

## Conclusions

Overall, these results may be relevant for processes of endogenous cartilage repair after blunt traumatization and, in a broader context, for the pathogenesis of osteoarthritis in general. They also encourage approaches towards *in situ *regeneration of cartilage defects based on local recruitment of chondroprogenitor cells. For transfer into novel therapeutic strategies, further questions have to be addressed: in case of a cartilage defect, matrices enabling CPC ingrowth have to be defined. We have observed an active outgrowth into a collagen type I hydrogel matrix (unpublished results) and similar results have been observed for PGA/PLA or PGA/PCL scaffolds [53]. The chondrogenic differentiation potential of CPC including interindividual variation, effect of cytokines [54], influence of aging, osteoarthritis and, as recently published, gender-related aspects like exposure to estrogen in women or testosteron in men [55] clearly need further investigation. The present study indicates that with respect to CPC recruitment, control of inflammatory processes is an important aspect that should be taken into consideration.

## Abbreviations

ACAN: aggrecan; ALPL: alkaline phosphatase; AMIC: autologous matrix induced chondrogenesis; ANOVA: analysis of variance; bFGF: basic fibroblast growth factor; BMP: bone morphogenetic protein; COL2A1: collagen type II; COL10A1: collagen type X; COMP: cartilage oligomeric matrix protein; CPC: chondrogenic progenitor cells; CTGF: connective tissue growth factor; DMEM: Dulbecco's modified Eagle's medium; HLA: human leukocyte antigen; HMGB-1: high mobility group box chromosomal protein 1; IGF-1: Insulin-like growth factor 1; IL-1β: interleukin-1 beta; IL-6: interleukin-6; ISCT: International Society for Cellular Therapy; LPL: lipoprotein lipase; MMP: matrixmetalloproteinase; MSC: mesenchymal stromal cells; MSCA-1: mesenchymal stem cell antigen-1; Oct-3/4: octamer-binding transcription factor; PCL: polycaprolactone; PDGF: platelet-derived growth factor; PGA: polyglycolic acid; PLA: polylactic acid; RAGE: receptor for advanced glycation end product; Runx2: runt-related transcription factor 2; SEM: standard error of the mean; SN: supernatant; SOX: Sry-related HMG box; Stro-1: a stromal cell precursor surface antigen; TGF-β1: transforming growth factor beta 1; TNF-α: tumor necrosis factor alpha.

## Competing interests

The authors declare that they have no competing interests.

## Authors' contributions

HJ carried out the scratch and outgrowth assays and performed statistical analyses. AW carried out the flow cytometric and differentiation analyses and performed statistical analyses. CH generated supernatant from cartilage, HR participated in the design and coordination of the study and provided study material. RB conceived and designed the study and drafted the manuscript. All authors assisted with interpretation of the data, helped to draft and/or revise the manuscript for intellectual content, and approved the final manuscript.

## Supplementary Material

Additional file 1 **Figure S1: Flow cytometric analysis of MSC (black) and CPC (gray) marker expression**. Open histograms represent marker-specific antibodies. Gray shaded histograms represent isotype control. CPC: chondrogenic progenitor cells; MSC: mesenchymal stromal cells. Click here for file

Additional file 2 **Figure S2: Marker expression profiles from MSC and CPC**. Both cell types were respectively stained with four markers in different combinations. The percentage of quadruple stained cells is depicted on the y-axis. Due to the varying patient collectives the number of different donors is indicated as n (range). CPC: chondrogenic progenitor cells; MSC: mesenchymal stromal cells. Click here for file

Additional file 3 **Figure S3: Flow cytometric and immunocytochemical analysis of CPC adhesion receptors and cytoskeleton expression**. **A**: Flow cytometric analysis of the integrin subunits α1, α2, α5 and α6. Open histograms represent marker-specific antibodies. Gray shaded histograms represent isotype control. Representative histograms of two independent experiments are shown. **B**: Cadherin-11 specific staining of chondrogenic progenitor cells (CPC). **C**: Fluorescence staining of actin stress fibers by Phalloidin-FITC (green), of Vinculin by immunocytology (red) and of the nuclei by DAPI (blue) in CPC. Click here for file

Additional file 4 **Figure S4**. Differentiation status of CPC before (t_0_) and after cultivation in basal (BM), adipogenic (AD), osteogenic (OD) or chondrogenic (CD, micromass culture) differentiation media for four weeks. **A**: Adipogenic differentiation: (1) house-keeping gene GAPDH; (2) lipoprotein lipase; (3) leptin. **B**: Osteogenic differentiation: (1) GAPDH; (2) alkaline phosphatase; (3) Osteocalcin; (4) runt-related transcription factor 2. **C**: Chondrogenic differentiation: (1) GAPDH; (2) collagen type II; (3) collagen type X; (4) cartilage oligomeric matrix protein; (5) aggrecan; (6) Sry-related HMG box (SOX9). CPC: chondrogenic progenitor cells. Click here for file

Additional file 5 **Figure S5: Effects of IL6 and PDGF on CPC migration**. The number of migrated cells without stimulation and stimulated with platelet-derived growth factor (PDGF) (10 ng/ml) and/or IL6 (1 ng/ml) is depicted. The values represent the mean ± SEM with *n *= 3. Statistical significance was evaluated by ANOVA followed by a Bonferroni multiple comparison post-test with *P < 0.05, **P < 0.01 and ***P < 0.001. CPC: chondrogenic progenitor cells. Click here for file

Additional file 6 **Figure S6: Effects of IL-1β and TNF-α on CPC outgrowth**. The number of outgrown cells without stimulation (Control) and stimulated with interleukin (IL)-1β (IL, 1 ng/ml) or tumor necrosis factor (TNF)-α (TNF, 10 ng/ml) is depicted in log scale. The values represent the mean ± SEM with *n *= 4. CPC: chondrogenic progenitor cells. Click here for file
