# Supplementary figures and images for: Interleukin-1 beta and tumor necrosis factor alpha inhibit migration activity of chondrogenic progenitor cells from non-fibrillated osteoarthritic cartilage
Source: Arthritis Res Ther. 2013 Sep 13;15(5):R119. doi: 10.1186/ar4299 (PMC3978440; doi:10.1186/ar4299)

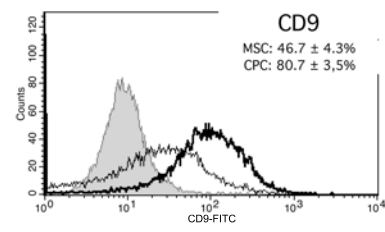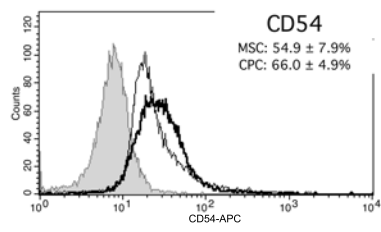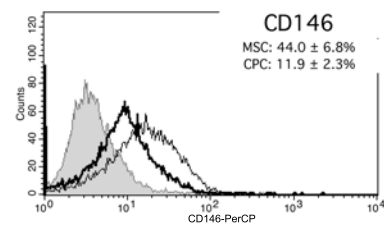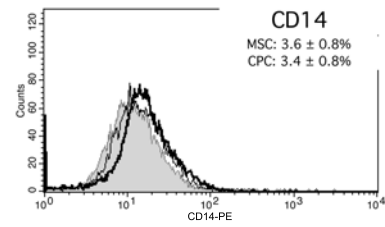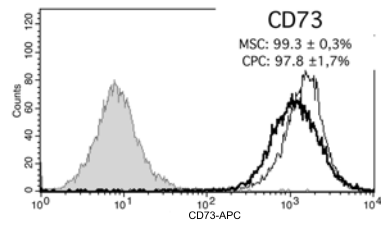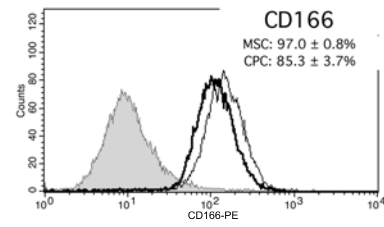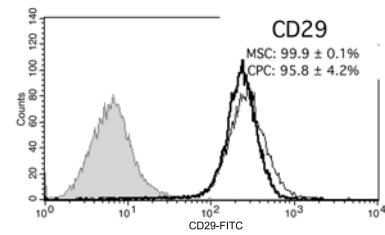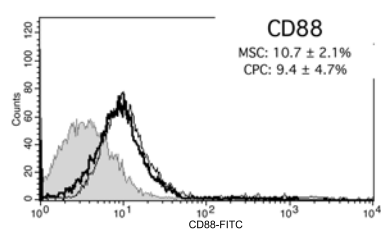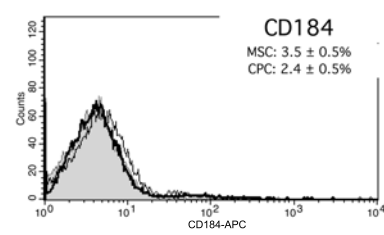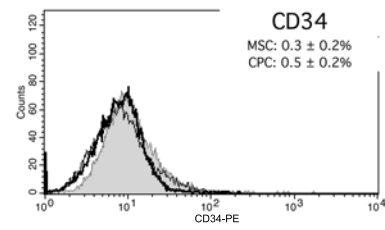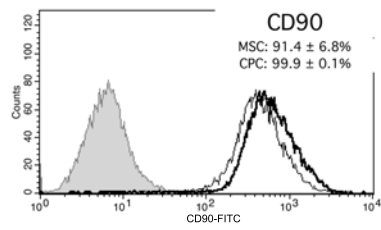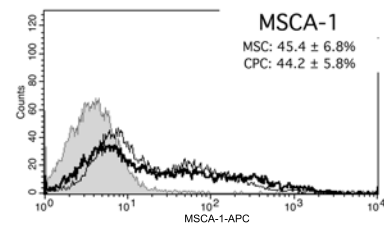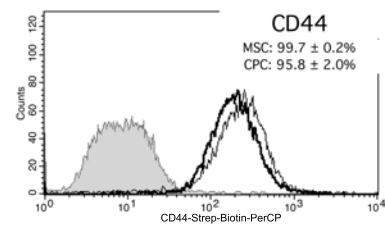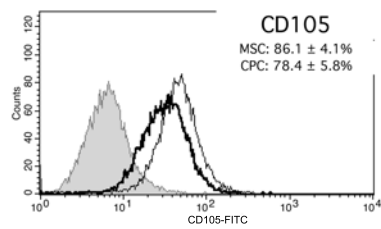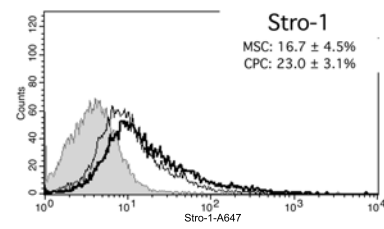

ge 2

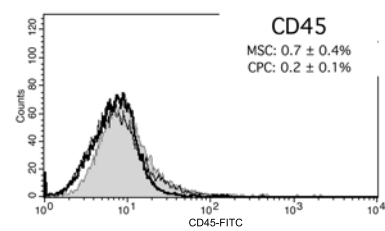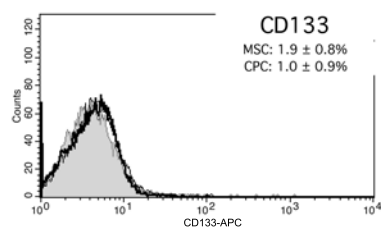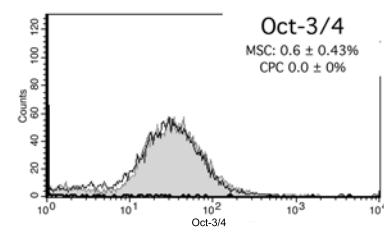

Supplement: Additional file 1 — Figure S1: Flow cytometric analysis of MSC (black) and CPC (gray) marker expression. Open histograms represent marker-specific antibodies. Gray shaded histograms represent isotype control. CPC: chondrogenic progenitor cells; MSC: mesenchymal stromal cells. [file ar4299-S1.PDF]

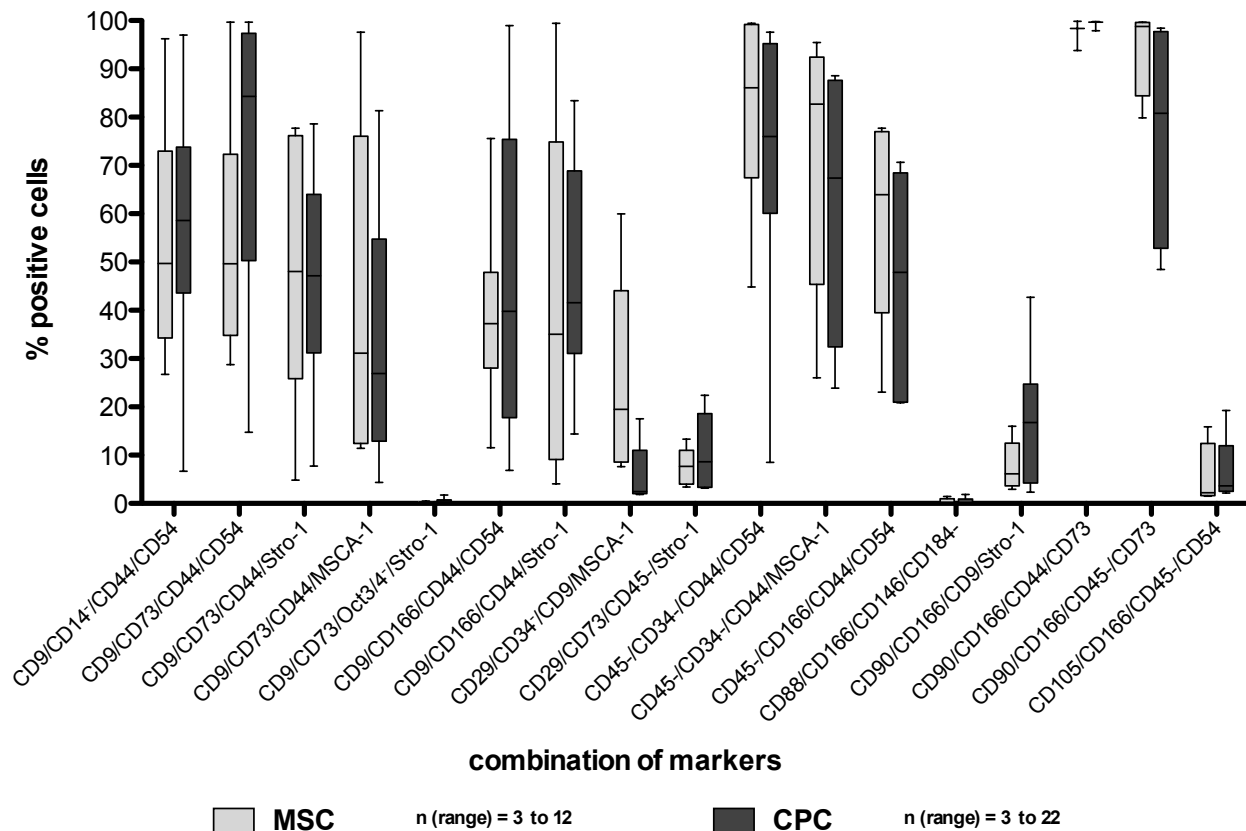

Supplement: Additional file 2 — Figure S2: Marker expression profiles from MSC and CPC. Both cell types were respectively stained with four markers in different combinations. The percentage of quadruple stained cells is depicted on the y-axis. Due to the varying patient collectives the number of different donors is indicated as n (range). CPC: chondrogenic progenitor cells; MSC: mesenchymal stromal cells. [file ar4299-S2.PDF]

**A**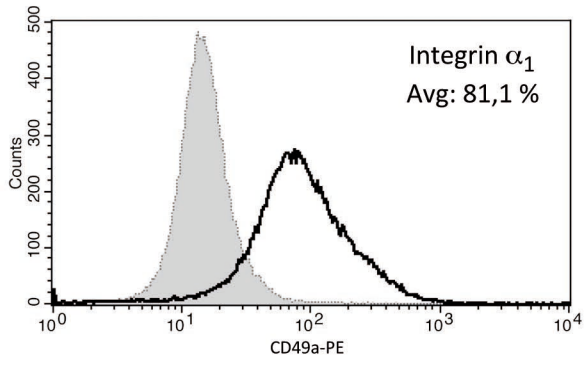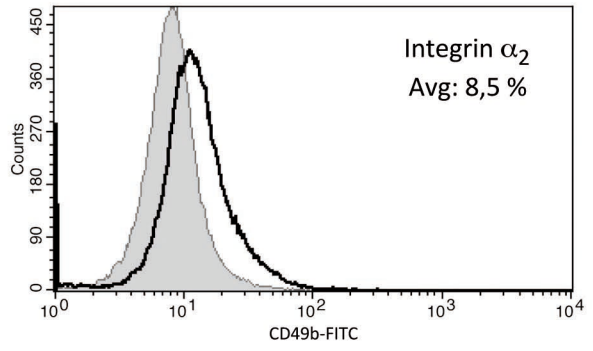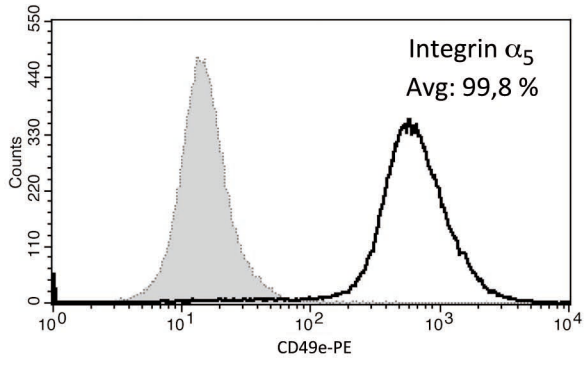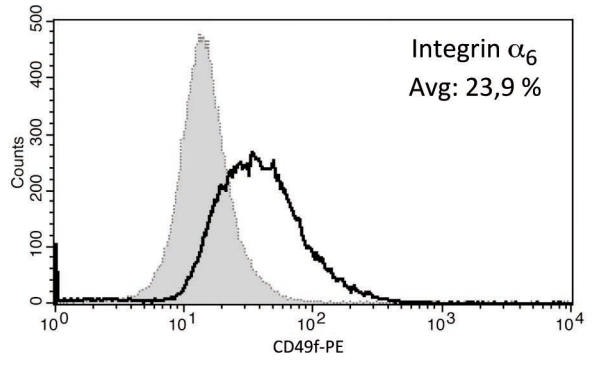**B**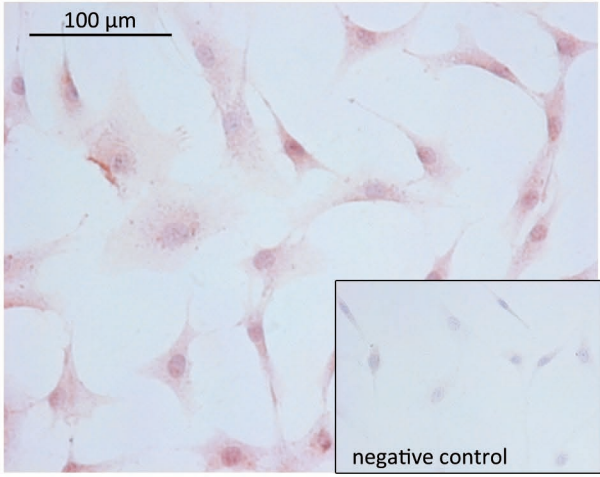**C**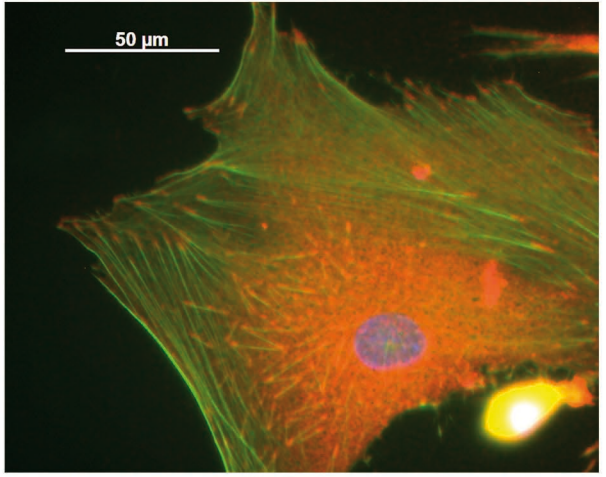

Supplement: Additional file 3 — Figure S3: Flow cytometric and immunocytochemical analysis of CPC adhesion receptors and cytoskeleton expression. A: Flow cytometric analysis of the integrin subunits α1, α2, α5 and α6. Open histograms represent marker-specific antibodies. Gray shaded histograms represent isotype control. Representative histograms of two independent experiments are shown. B: Cadherin-11 specific staining of chondrogenic progenitor cells (CPC). C: Fluorescence staining of actin stress fibers by Phalloidin-FITC (green), of Vinculin by immunocytology (red) and of the nuclei by DAPI (blue) in CPC. [file ar4299-S3.PDF]

**A**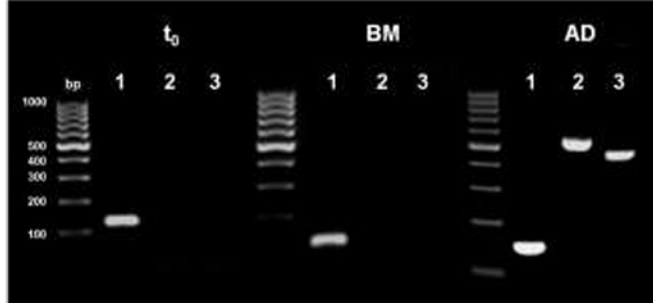**B**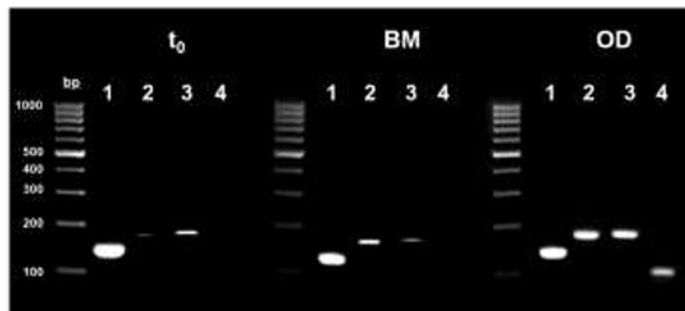**C**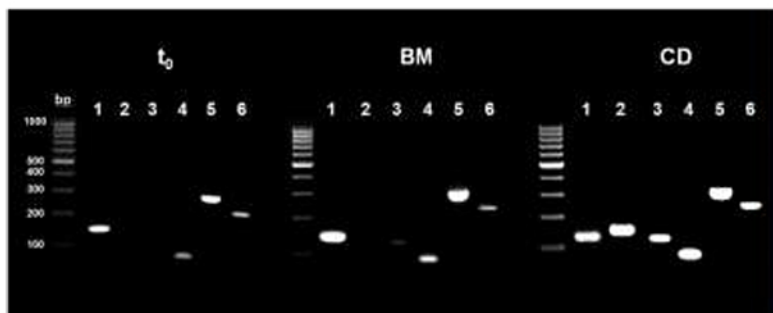

Supplement: Additional file 4 — Figure S4. Differentiation status of CPC before (t0) and after cultivation in basal (BM), adipogenic (AD), osteogenic (OD) or chondrogenic (CD, micromass culture) differentiation media for four weeks. A: Adipogenic differentiation: (1) house-keeping gene GAPDH; (2) lipoprotein lipase; (3) leptin. B: Osteogenic differentiation: (1) GAPDH; (2) alkaline phosphatase; (3) Osteocalcin; (4) runt-related transcription factor 2. C: Chondrogenic differentiation: (1) GAPDH; (2) collagen type II; (3) collagen type X; (4) cartilage oligomeric matrix protein; (5) aggrecan; (6) Sry-related HMG box (SOX9). CPC: chondrogenic progenitor cells. [file ar4299-S4.PDF]

Number of migrated cells

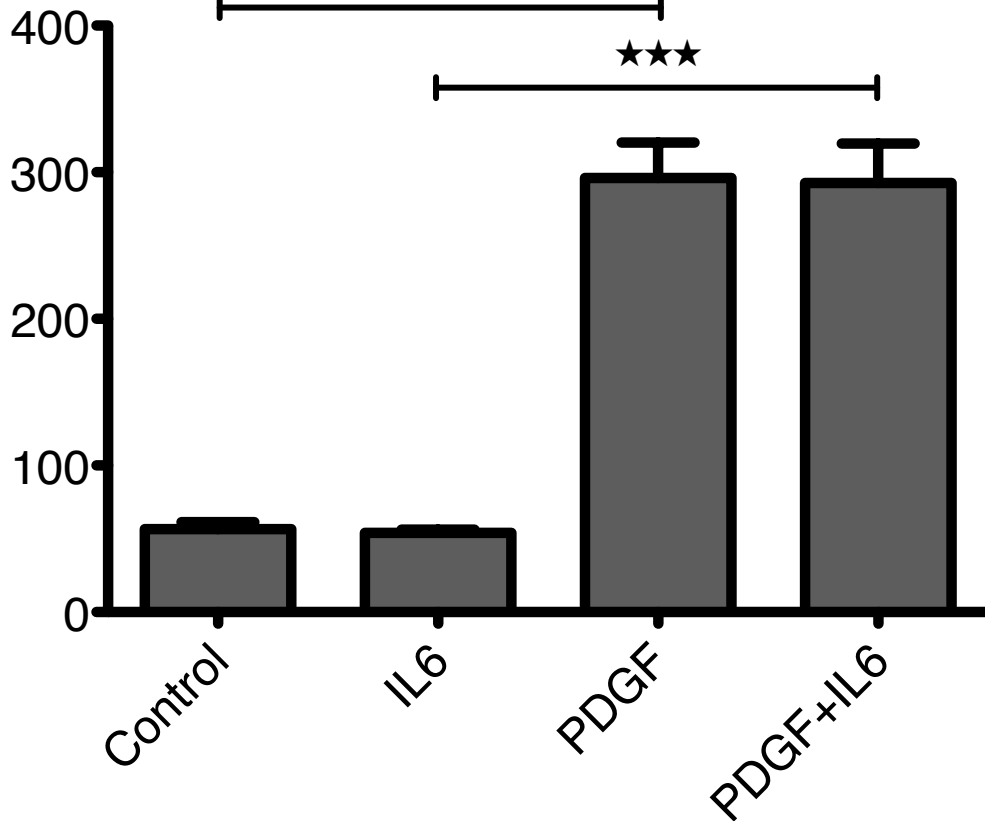

Supplement: Additional file 5 — Figure S5: Effects of IL6 and PDGF on CPC migration. The number of migrated cells without stimulation and stimulated with platelet-derived growth factor (PDGF) (10 ng/ml) and/or IL6 (1 ng/ml) is depicted. The values represent the mean ± SEM with n = 3. Statistical significance was evaluated by ANOVA followed by a Bonferroni multiple comparison post-test with *P < 0.05, **P < 0.01 and ***P < 0.001. CPC: chondrogenic progenitor cells. [file ar4299-S5.PDF]

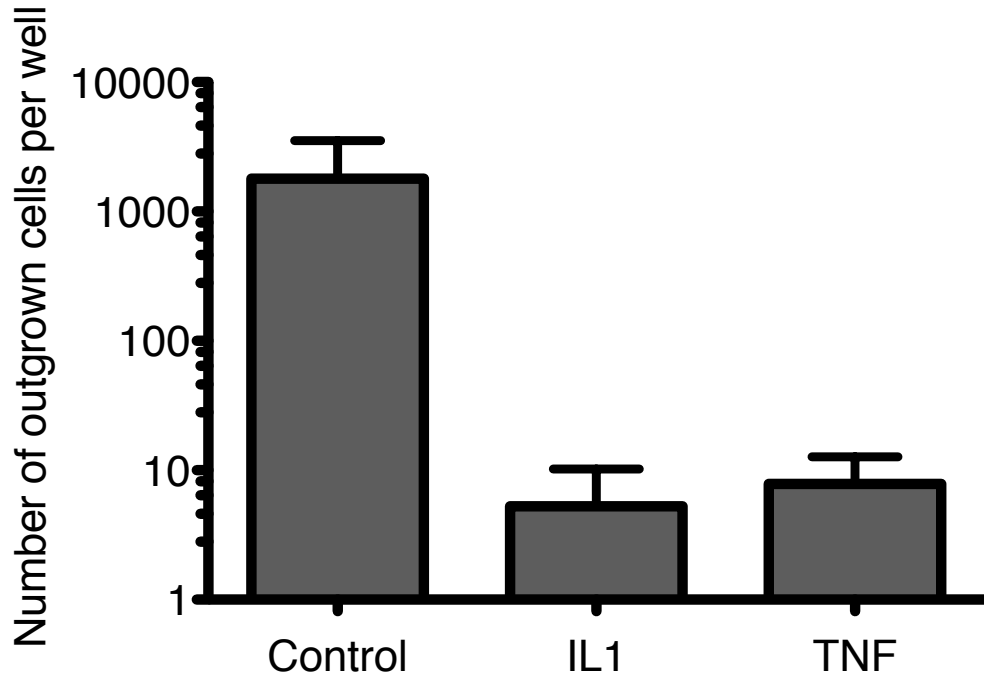

Supplement: Additional file 6 — Figure S6: Effects of IL-1β and TNF-α on CPC outgrowth. The number of outgrown cells without stimulation (Control) and stimulated with interleukin (IL)-1β (IL, 1 ng/ml) or tumor necrosis factor (TNF)-α (TNF, 10 ng/ml) is depicted in log scale. The values represent the mean ± SEM with n = 4. CPC: chondrogenic progenitor cells. [file ar4299-S6.PDF]
